# Supplementary material for: Role of Intestinal Inflammation and Permeability in Patients with Acute Heart Failure
Source: Medicina (Kaunas). 2023 Dec 20;60(1):8. doi: 10.3390/medicina60010008 (PMC10821136; doi:10.3390/medicina60010008)
Supplement: Supplementary file 1 [file medicina-60-00008-s001.zip › medicina-2666035-supplementary.pdf]

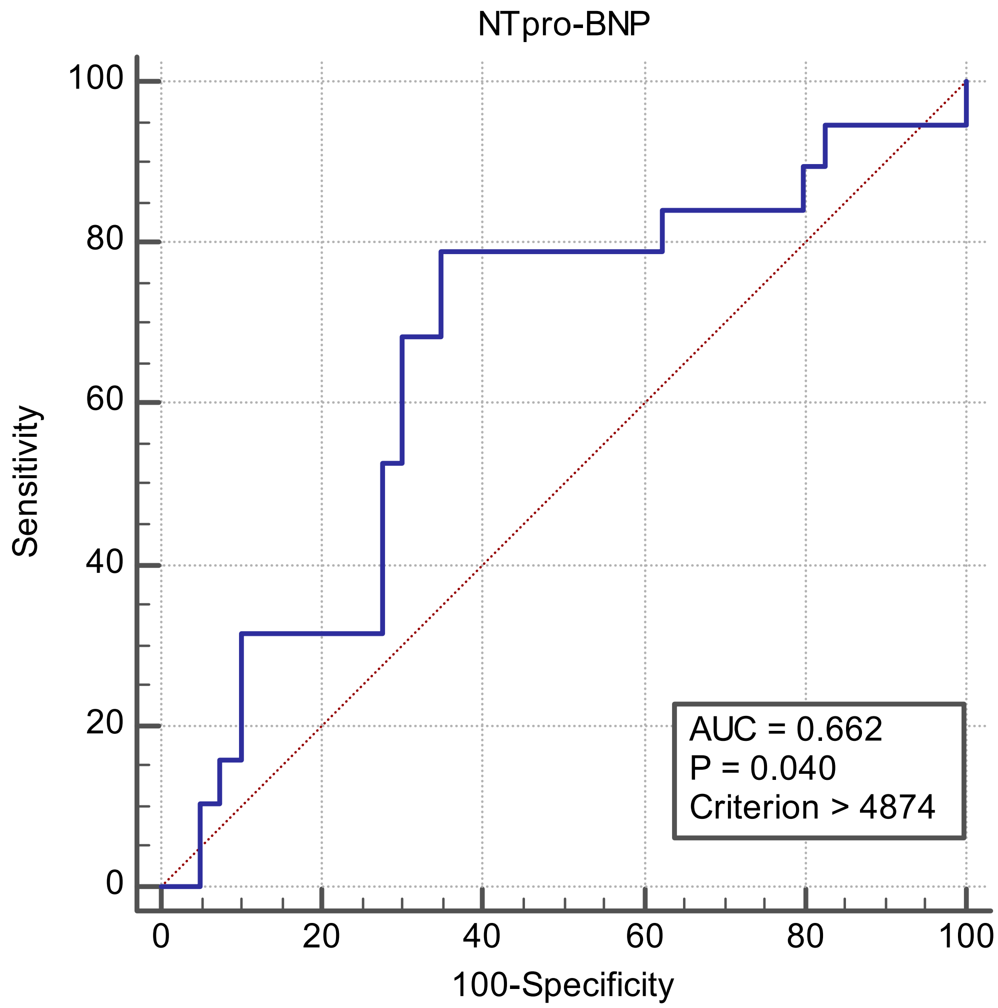

Criterion	Sensitivity	95% CI	Specificity	95% CI
§NT-proBNP>4874 pg/ml	78.95	54.4 – 93.9	65.00	48.3 – 79.4

§Youden Index J cut-off value. All the patients in the study cohort had baseline NT-proBNP values above the standard reference value

Supplementary Figure S1 – Receiver Operating Characteristics (ROC) analysis of NT-proBNP values and the study endpoint (Cumulative death/readmission at 90 days).

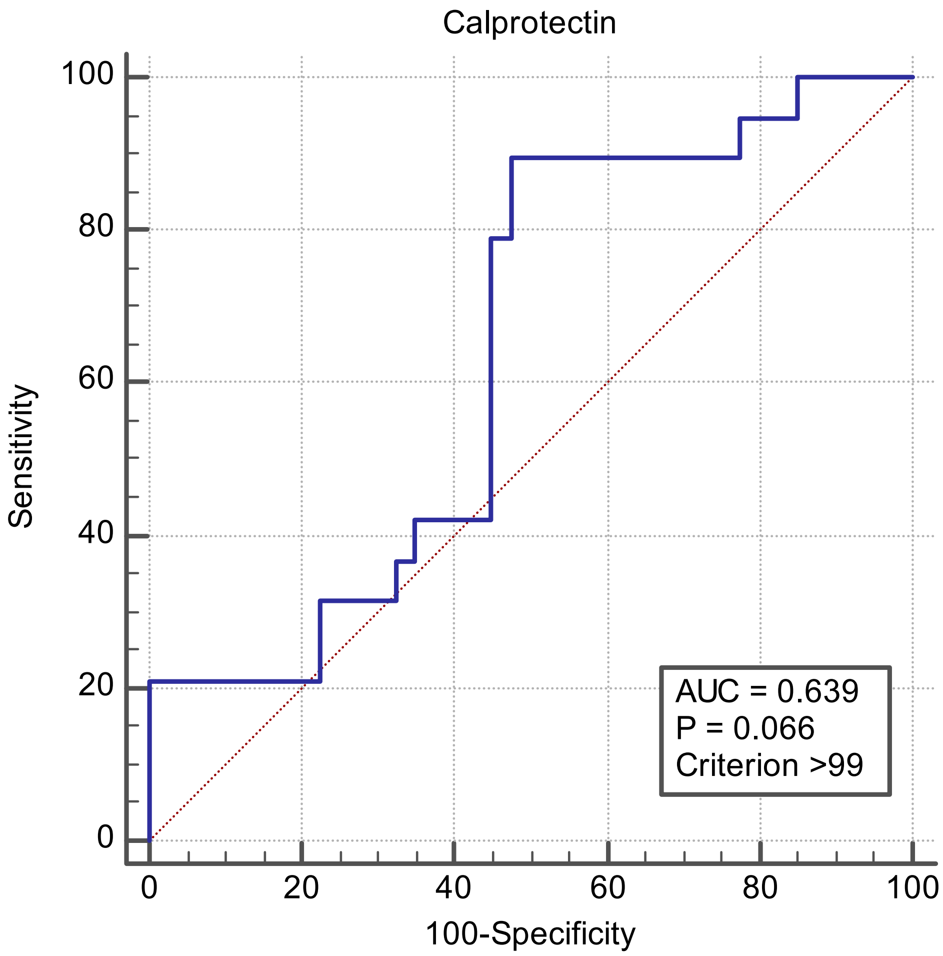

Criterion	Sensitivity	95% CI	Specificity	95% CI
*Calprotectin >50 µg/g	89.47	66.9 - 98,.7	32.50	18.6 – 49.1
§Calprotectin >99 µg/g	89.47	66.9 – 98.7	52.50	36.1 – 68.5

*Standard reference value

§Youden index J cut-off value

Supplementary Figure S2 – Receiver Operating Characteristics (ROC) analysis of Calprotectin values and the study endpoint (Cumulative death/readmission at 90 days).

Supplementary Table S1. Multivariate logistic regression analysis of variables correlated to the main combined outcome. Separate models were assessed for standard reference value of calprotectin, and for ROC Youden index J derived cut-off value.

	**Beta**	**SE**	**Walds statistic**	**P value**	**OR [95% CI]**
FC>50 µg/g	1.823	.874	4.351	0.037	6.192 [1.12 – 34.3]
NT-proBNP> 1249 pg/ml	2.208	.698	10.013	0.002	9.093 [2.31 – 35.7]
Constant	-2.927	.834	12.318	<0.001	.054
FC>50 µg/g	2.208	.698	10.013	0.002	9.09 [2.32 – 35.7]
NT-proBNP> 1249 pg/ml	1.823	.874	4.351	0.037	6.19 [1.12 – 34.3]
Constant	-2.927	.834	12.318	<0.001	.05

Abbreviations: FC: fecal calprotectin¸ NT-proBNP: N-terminal fragment B-type natriuretic peptide; SE: standard error; Sign: significance; OR: Odd Ratios, CI: Confidence interval.
